# Study protocol of group antenatal care implementation at a public women’s hospital in Brazil

**DOI:** 10.1371/journal.pone.0326084

**Published:** 2025-06-25

**Authors:** Larissa Rodrigues, Fernanda Garanhani Surita, Maria Laura Costa, Odette del Risco Sánchez, Ysabelle Cristina Cardoso Marinato, Nicole Julie Scarpino

**Affiliations:** 1 Women’s Health and Newborn’s Health Area, School of Nursing, Universidade Estadual de Campinas (UNICAMP), Campinas, São Paulo, Brazil; 2 Department of Obstetrics and Gynecology, School of Medical Science, Universidade Estadual de Campinas (UNICAMP), Campinas, São Paulo, Brazil; 3 Postgraduate Program in Obstetrics and Gynecology, School of Medical Science, Universidade Estadual de Campinas (UNICAMP), Campinas, São Paulo, Brazil; 4 Bachelor’s Program in Nursing, School of Nursing, Universidade Estadual de Campinas (UNICAMP), Campinas, São Paulo, Brazil; University of Campinas, BRAZIL

## Abstract

The antenatal care is an important service offered to women during pregnancy that aims to guarantee the mother and fetus’s health. In Brazil, although the coverage of this service is high, the outcomes need to be improved. This study aims to implement a pilot study of a group antenatal care among pregnant women in follow up at a Woman´s Hospital of Universidade Estadual de Campinas, a public teaching hospital referral in women’s health. This randomized clinical trial will be conducted. The study group will be composed of a maximum of 15 women that will be followed in a group antenatal care with 11 meetings every 4 weeks or earlier, during all pregnancy period, while control group will receive the individual antenatal care. Sociodemographic data, quality of life, anxiety and depression will be assessed in women from both groups and women’s satisfaction and experience among the intervention group will be evaluated. A 5% significance level was adopted for quantitative analysis and the sample size was calculated based on ambulatory’s flow. A positive result among the intervention group is expected, mainly in providing a better antenatal experience for women and their knowledge of maternal and child health. This study was approved by the Ethics and Research Commission of UNICAMP number of approval: 25333119.2.0000.5404 and the Brazilian Registry of Clinical Trials (REBEC): RBR-8xcjmf UTN code: U1111-1243–6241. Date: jan.2025, version 1.

## Background

The antenatal care is a service with worldwide relevance that offers a follow up of maternal and fetal conditions from pregnancy diagnose to childbirth and happens in one of the three care levels (primary, secondary and tertiary) according to women’s risk [1]. In Brazil, through the Unified Health System (SUS) (a public health system offered to all population), a minimum of 6 individual medical consultations for each woman was established with laboratory and image exams following a protocol according to gestational age and a medical consultation postpartum [2].

A new antenatal care group model is being developed with good outcomes. The main difference compared to the traditional model is the group approach against the individual care. The group antenatal care has been implemented in United States of America [3,4] since 1995 and receives the name of ‘*’Centering Pregnancy*.” In this new model, the care is provided by an obstetric nurse or physician to a group composed by eight to twelve pregnant women with similar gestational age and the group gather from eight to ten times during pregnancy in scheduled meetings with duration to 90–120 minutes.

All provided care are realized at this group environment. The physical evaluation routine, education and woman support with a development of guide discussions about themes related to pregnancy, possible complications, woman and newborn care, contraception and familiar relationship, promoting integral care to these women [1,3–8].

The physical exam is performed separately only by a folding screen, in which the physician auscultates the fetal heartbeat, determines the uterine fundal height measurement and if necessary, a gynecological exam. The physician asks if the woman has any question and request authorization to share the questions with the group. Meanwhile, in the group environment, the pregnant women take turns with the responsibility of evaluating and writing their own weight and blood pressure (using digital device, checked by the medical team) [1,3–8].

The researches have been showing good outcomes to subjective questions about woman experience during group participation, like trend in early breastfeeding, smaller hospitalization rate in Neonatal Intensive Care Unit (NICU) and decrease in preterm labor [4,9]. In women with diabetes, there was a smaller necessity to drug treatment and women who needed insulin used less than half of doses comparing to women that received the traditional care. Preterm labor decreased and an increase in breastfeeding were observed among women with low income and Afro-American and black women, showing that the new model is applicable among different pregnant profiles [5,6,10,11].

The group antenatal care optimizes the health professional routine by reducing the professional’s work time and the need to repeat the information to every patient individually [2,12] and the implementation of this new antenatal care model can attend underlying SUS principles, specialty in relation to comprehensiveness and universality attending.

In Brazil, the coverage of antenatal care in south region achieves 98%, but the quality and maternal-child outcomes need to be improved, and this new strategy shows to be a good alternative to reach greater quality and women’s preparation to the post labor period, promoting better outcomes [7,13,14].

Although group antenatal care with high fidelity to principles in several low- and middle-income settings has shown better perinatal outcomes compared to traditional care [15], more research is needed to describe potential differences in outcomes between group and traditional antenatal care [16].

The current body of literature still supports group antenatal care as a potential tool to decrease racial inequities in antenatal access, quality of care, and maternal mortality. Furthermore, pregnant women can be benefited by participating in the antenatal care group, but more studies are necessary to determine the benefits of this antenatal care approach [1,16].

In Brazil, there are still no studies showing these differences. Therefore, this study aims to: 1) Implement a pilot project of group antenatal care in one of the antenatal care outpatient clinics from the Woman´s Hospital of Universidade Estadual de Campinas (UNICAMP), a public university in Campinas, Brazil; 2) Compare maternal and perinatal outcomes between the women from antenatal group care and the traditional antenatal care; 3) Understand the experience of women followed in antenatal group care and 4) Observe and analyze the meeting’s dynamic of antenatal group care.

## Methods/design

### Study type

Randomized clinical trial.

### Setting

The study will be conducted at the Antenatal Outpatient Clinic of the Center for Comprehensive Care for Women’s Health (CAISM), which is part of the Hospital complex of the Universidade Estadual de Campinas (UNICAMP).

The unit is located in a public Hospital, dedicated to health care, teaching and research. This Hospital is a reference unit for more than 40 municipalities in the region. It offers tertiary hospital care, as well as outpatient care in the areas of gynecology, obstetrics, neonatology and oncology.

The CAISM antenatal outpatient clinic treats pregnant women referred from Primary Health Care who present with gestational risk situations such as: hypertensive disorders, diabetes, thyroid pathologies, obstetric history with complications, among others, or pregnant women with low risk who are divided according to the situation or concomitant disease on different days of the week. The proposed study will begin with the antenatal care that takes place on Thursday, where women with low risk pregnancies are concentrated.

### Inclusion and exclusion criteria

The inclusion criteria are pregnant women with 18 years old or more; gestational age (GA) less than 20 weeks of gestation and antenatal care at the study setting. - The exclusion criteria is professional indication validated by a professor to individualize care (will be sent to the indicated professionals or sectors, e.g., hospitalization, another outpatient clinic, etc.).

### Intervention

Initial training will be provided prior to implementation of the intervention so that all stages are discussed and understood by all members of the delivery team. Topics related to the eleven planned meetings, the needs of women during antenatal and postpartum follow-up, and the references and protocols used for individual and group antenatal models will be discussed. Discussions and training will also be provided on the intervention delivery process and its evaluation, as well as on the characteristics of the interventionists [17]. In addition, an intervention manual was created with the script for each meeting and the materials used in each time allocated to the planned activities.

### Randomization and masking method

After accepting to participate in the research and signing the informed consent form, the researcher will randomly select the assigned group. We will use a computerized randomizer. A random sequence will be generated by a computer software (http://www.random.org/) and will be followed for the allocation of participants into the experimental and control groups. A sealed envelope containing the randomization form previously filled out in the Intervention Group or Control Group, which will be opened and read with the patient.

The study will not be masked, since the pregnant woman and the researcher will know whether the patient belongs to the IG or CG.The Intervention Group (IG) is going to be composed by a maximum of 15 pregnant women. The group will be attending in 11 meetings with approximately two-hour duration each, every 4 weeks or earlier, if necessary, with the following themes discussions:

Nutrition and physical activity during pregnancy; Most common management complaints and signs of complications; Social and social security rights and mental health; Reproductive planning and sexuality; Childbirth preparation guidelines, warning signs and maternity visit; Breastfeeding; Gestational complications and preparation for trousseau; Cognitive and physical development of the child; Nutrition in the fourth quarter; Postpartum follow-up routines and group closure.

### Follow-up

The pregnant women will be followed during all pregnancy period (with one postpartum review) through scheduled visits in every 4 weeks or earlier (according to women necessity), with a minimum of 10 group consultations. The women can come to the hospital other days for laboratory exams, but the attending will always happen in the group.

At each meeting, for each participant, the meeting information will be recorded on an antenatal card, with information like: attendance data, meeting number, gestational age, weight, blood pressure, uterine height, fetal heart rate, fetal movements, fetal presentation, edema, time to return to the clinic, local and professional care. The Control Group (CG) will follow individualized antenatal care according to the service routine, which consists of medical and nursing visits scheduled every 4 weeks or earlier, if necessary.

The data collection process will occur during all antenatal care period. After the inclusion in the research and before starting the antenatal care, the participants (from both groups) will answer a questionnaire about social and demographic questions. Then, will participate in a semi direct individual interview with themes related to women’s expectations about antenatal care and will answer, individually, World Health Organization Quality of Life (WHOQOL brief) [18], State-Trait Anxiety Inventory (IDATE) [19] and Edinburgh [20] scale questionnaires.

Women allocated in intervention group, will participate in focus group also with themes related to women’s expectations about antenatal care, at the first attending of group antenatal care. To evaluate the intervention’s effectiveness, at the finals attending of antenatal care (preferably after 38 weeks of pregnancy) or in postpartum visit, all pregnant women included in the study will answer again the WHOQOL, IDATE and Edinburgh scale questionnaires and a new individual interview will be performed. In all intervention group meetings, pre and post-tests will be carried out on the experience and satisfaction regarding the central theme of each meeting. A new focus group will be performed with both groups (intervention and control).

Women who withdraw or have a medical recommendation to leave IG will be discontinued and these will also participate in an interview with open questions to assess the reasons for giving up on group antenatal care.

Data about pregnancy, maternal and perinatal outcomes will be assessed by medical record review. Strategies like phone call reminder for each appointment and evaluation of women’s satisfaction will be adopted to improve the adherence in study.

### Procedures and techniques

The procedures will be done by the research team. The physical exams will be performed by an obstetrics nurse or obstetrics medical doctor that will participate in the group during all attending period. While a woman is in physical exam, the others will take turns with the responsibility of evaluate and write their own weight and blood pressure (using digital device confirmed by the medical team). After the physical exam, the women will be able to make questions and participate in the group discussion. Although the themes were previously chosen for each meeting, the group will be conducted according to women necessity and other themes can arise.

The final interviews among focus groups and questionnaires will be performed by a researcher that did not participate in the group.

### Sample size

**Fig 1:**
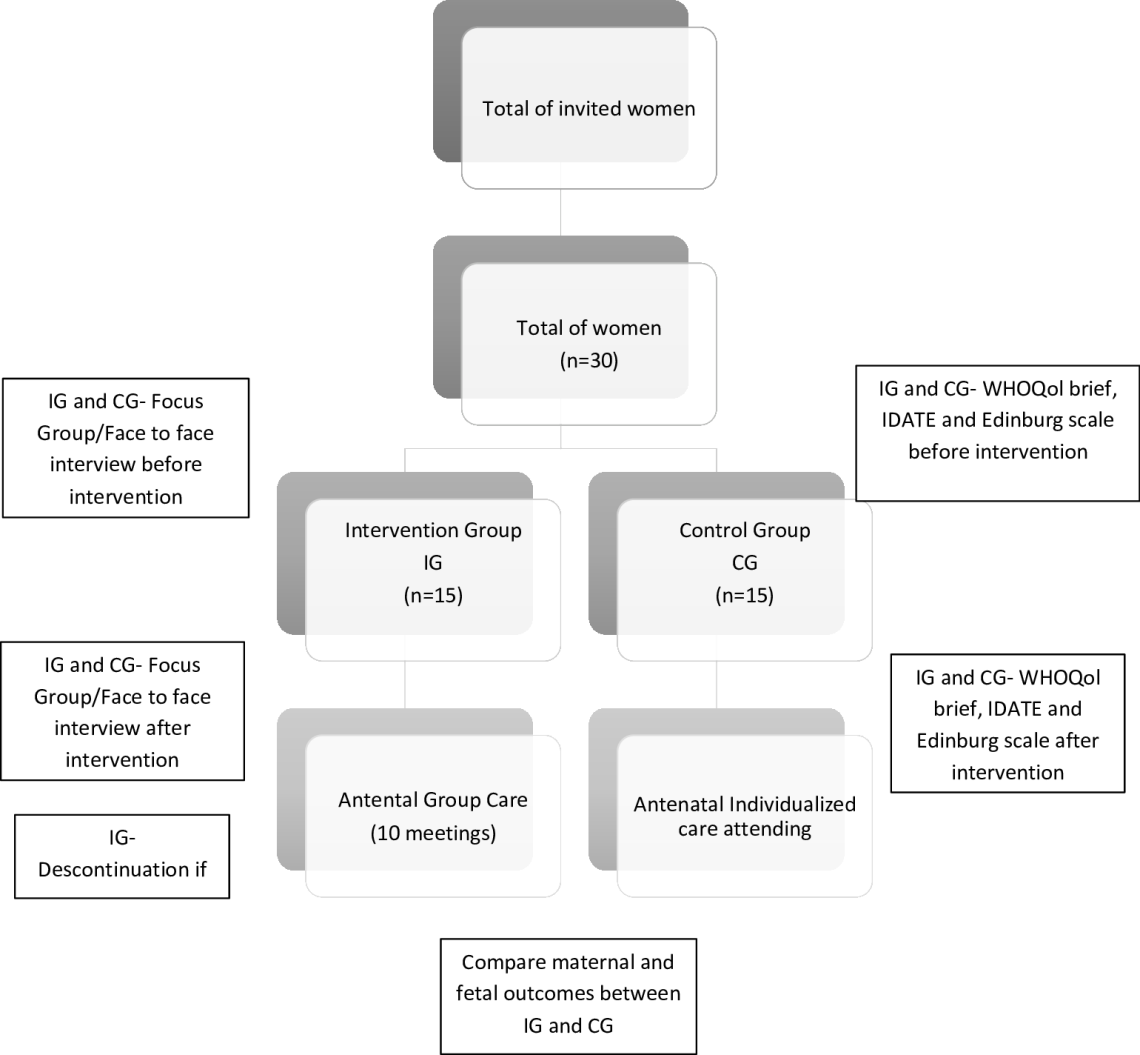
SPIRIT flowchart.

The sample size determined was 12–15 participants per group, taking into account sample size recommendations for pilot studies [21,22]. The sample will consider new cases attended at the research setting in each period. Fig illustrates the process in SPIRIT flowchart [Fig 1].

### Statistical analysis

The quantitative data will be saved in a spreadsheet of Microsoft Excel® Software and will be stored for a five-year period. A descriptive analysis will be conducted using measures of central tendency and dispersion for quantitative variables and frequencies and percentages for categorical variables. The groups and time periods will be compared with regard to quantitative outcomes using the unpaired or paired Student’s t-test, Mann-Whitney test or paired Wilcoxon test, according to the data distribution.

Data distribution will be assessed using the Shapiro-Wilk test. Pearson’s chi-square, Fisher’s exact or McNemar’s tests will be used for categorical outcomes. The Bonferroni correction [23] will be applied to the significance level according to the number of comparisons made for each outcome variable. The significance level was set at 5% and the program *Statistical Analysis System* (SAS), version 9.4 will be used.

### Qualitative analysis

The qualitative data from individual interviews and focus group will be recorded and transcribed, then anonymous encoded and will be storage in two ways: At NVIVO14 Software and in the research pen drive, for a five-year period. Qualitative data will be analyzed by themes in the following steps: 1) Text edition to transcription maintained the originals traits of participants; 2) Listen and rereading of transcriptions; 3) Comments and registered impressions in front of this rereading, writing at right bank of transcribed text; 4) Categorization and subcategorization of information; 5) Presentation and discussion of material to pairs; 6) Categories definition as result of material enhancement and data validation by pairs [24] .

All material used for data collection will be stored in two ways: on the NVIVO 14 software platform and in the UNICAMP data repository (REDU- https://www.sbu.unicamp.br/sbu/repositorio-de-dados-de-pesquisa-da-unicamp/). This article has followed the SPIRIT guidelines for its elaboration [25] [S1 File].

### Plan for translation and dissemination of scientific knowledge

During the IG meetings, materials (leaflets with QR codes) will be distributed to popularize scientific knowledge on the various topics covered, enabling access and sharing of the topics discussed at any time. This way, women will be able to review information, reflect and formulate questions for the next meetings, share and talk with family members and support networks in general. Professionals working in CAISM’s antenatal care clinics will be informed about the results of the intervention at the end of the implementation of the pilot project, and dissemination to the scientific community will be done through the production and publication of scientific articles.

### Quality control

Procedures will be adopted for quality control, such as: subject identification numbers to prevent data loss or confusion; review of completed forms, organization of instruments, and verification of the coding of textual responses; data will be entered into the database by two different data entry clerks to avoid data loss or typing errors.

### Reports the status and timeline of the study

A) initial training for the intervention team will be held in March and June 2025; B) participant recruitment has not started and is scheduled to take place between May 01 and June 30, 2025; C) data collection will be completed by January, 2026; D) Qualitative data analysis and validation with peer researchers, using NVIVO14 and Quantitative analysis with support from the university’s statistics service and E) results are expected by March, 2026.

### Ethics approval and consent to participate

The Resolution 466/12 [26] of the National Health Council are being followed. Women with 18 years old or more will be invited to the study and will be included after read, understand and sing the consent form. The researcher will explain the consent form and will inform that they can withdraw the study without any harm or loss in antenatal care. The study data will be used for the sole purpose of this study, and a commitment made to maintain confidentiality as to the identity of the interviewees in the disclosure of the data. This study was approved by the Ethics and Research Commission of UNICAMP under number CAAE: 25333119.2.0000.5404 [S4]. It has also been entered into REBEC: RBR-8xcjmf.

## Discussion

With the implementation of this intervention, we expect to provide a better antenatal experience for women under our care and thereby meet the WHO and ACOG recommendations for this service [7,8]. We understand the best experience in the sense of being more prepared to manage the pregnancy and postpartum period with greater strengthening for self-care, baby care and empowerment for reproductive choices such as new pregnancies and better contraceptives to be used.

We also expect a positive result in IC related to topics about maternal and child health. Is expected to find a higher quality of life and low rates of anxiety and depression among women in IC when compared with CG. About issues related to child health, is expected low rate of preterm labor, and higher burden among children who mother participates in group antenatal care and a trend in early breastfeeding. With these results, we also believe in an impact on cost reduction [11,14].

Our hypotheses are based in studies developed by Ickovics’ [3,4] in USA, that compared the two models of antenatal care (traditional and in group) and highlighting the benefits of antenatal group care, as: greater knowledge about pregnancy and post labor care, better ability on familiar relationship and more time with the health professionals (about 20 hours against 2 hours in individual attending)

## Potential limitations and mitigation strategies

The main potential biases of the study are those commonly associated with randomized clinical trials. During the discussions on the study design, several precautions were taken to minimize the risks, as outlined below:

Selection bias: To minimize this risk, we will rely on computer-generated random numbers. Performance bias: To further minimize this, sequentially numbered, opaque, and sealed envelopes will be used. Performance bias: The study will not involve blinding or masking. The authors will discuss potential biases in the process after the completion of the pilot study. Detection bias: There will be no blinding in the outcome assessment, but the authors believe that the outcome cannot be influenced by the lack of blinding.

Some challenges, such as late initiation of prenatal care and the cultural preference for traditional individual prenatal consultations, are anticipated during the recruitment process. To address this, awareness and information about the project will be shared with healthcare professionals to refer women to the project.

Loss to follow-up and participant non-adherence are potential issues, as there may be dropouts or refusals due to the belief that individual consultations are more appropriate, given that this is the traditional model used nationwide. To address this, several strategies will be implemented, including appointment reminders via phone (with patient consent), raising awareness among participants at the end of each session about the importance of the next topic, and reviewing the study objectives at the conclusion of each session.

## Supporting information

S1 Data Management and sharing plan. (DOCX)

S2 File SPIRIT_Fillable-checklist-2013. (PDF)

S3 File Fillable-SPIRIT-Outcomes-2022-Checklist. (PDF)
