# Supplementary material for: Study protocol of group antenatal care implementation at a public women’s hospital in Brazil
Source: PLoS One. 2025 Jun 25;20(6):e0326084. doi: 10.1371/journal.pone.0326084 (PMC12192027; doi:10.1371/journal.pone.0326084)
Supplement: S1 Data — (DOCX) [file pone.0326084.s001.docx]

# Data management and sharing plan

# Element 1: Data Type

**A.Types and amount of scientific data expected to be generated in the project:**

*Data to be generated from this project include responses of questionnaires(World Health Organization Quality of Life (WHOQOL brief), State-Trait Anxiety Inventory (IDATE) and Edinburgh scale) and speeches obtained through individual interviews and focus groups captured through digital audio recordings. We will collect data from 30 participants across dois timepoints e em outros 11 momentos possíveis.*

# B.Scientific data that will be preserved and shared, and the rationale for doing so:

*The University where the research will be developed has a policy of sharing raw data through an institutional repository. Thus, the authors decide to share the de-identified raw data as follows: The de-identified survey responses will be shared. Digital recordings at the individual or collective level will not be shared publicly due to the difficulty of masking the identity of the participants, but their transcripts will be anonymized/de-identified and shared.*

# C.Metadata, other relevant data, and associated documentation:

# *Metadata will include research instruments (questionnaires and scripts), researchers' field diaries, study protocol and other supporting documentation.*

# Element 2: Related Tools, Software and/or Code:

The data will be shared in Word or PDF files. No specialized software or tools are required to access the data files.

# Element 3: Standards:

# That no consensus standards exist.

# Element 4: Data Preservation, Access, and Associated Timelines

1. **Repository where scientific data and metadata will be archived:**

Data and metadata will be made available through *he UNICAMP data repository (REDU - https://www.sbu.unicamp.br/sbu/repositorio-de-dados-de-pesquisa-da-unicamp/)*.

# How scientific data will be findable and identifiable:

Data and metadata will be assigned a unique Digital Object Identifier (DOI) and a formal study citation by the repository.

# When and how long the scientific data will be made available:

All de-identified data will be available, and will remain available indefinitely.

# Element 5: Access, Distribution, or Reuse Considerations

**A.Factors affecting subsequent access, distribution, or reuse of scientific data:**

*Data will be shared in a two-tier distribution system. Any data that can be de-identified will be shared publicly. Data with specific confidentiality risks, such as individual or collective digital recordings, will not be shared publicly due to the difficulty of concealing the identity of participants.*

# B.Whether access to scientific data will be controlled:

*De-identified data will be freely available on the repository website.*

# C.Protections for privacy, rights, and confidentiality of human research participants:

*All direct participant identifiers will be removed from data prior to sharing. Study participants will be asked to consent to widespread data sharing with the research community based upon recommendations of the local IRB and approval of the consent document. The institution will determine whether the de-identified individual-level data are subject to the Certificate of Confidentiality policy, and if so, will protect accordingly and ensure that recipients of the data are aware of the protection.*

# Element 6: Oversight of Data Management and Sharing:

*The Research Data Management Committee (CGDP) is responsible for supporting and promoting Unicamp's institutional research data policy. The CGDP is also responsible for managing the Research Data Repository (REDU), created by CCP Resolution 006/2020, and qualified as an official instrument responsible for storing digital content in the form of software and raw research data. Composition of the Research Data Management Committee for the 2024/2026 term (CCP Resolution 004/2024): Claudia Maria Bauzer Medeiros – President (Technology/IC), Rodolfo de Carvalho Pacagnella (Biomedical/FCM), Benilton de Sá Carvalho (Exact Sciences/IMECC), Rodrigo Esteves de Lima Lopes (Humanities and Arts/IEL), Marcelo Mendes Brandão (COCEN), Breno Bernard Nicolau de França (DETIC), Márcio Souza Martins (SBU).*

*Other information: https://prp.unicamp.br/comissoes/gestao-de-dados-de-pesquisa/comissao*
